# COVID-19 related psychological distress and fears among mothers and pregnant women in Saudi Arabia

**DOI:** 10.1371/journal.pone.0256597

**Published:** 2021-08-24

**Authors:** Abdulkarim M. Meraya, Mamoon H. Syed, Ayesha Yasmeen, Amal A. Mubaraki, Hadi Dhafer Kariry, Wafaa Maabouj, Dhaifallah Moraya, Hafiz A. Makeen

**Affiliations:** 1 Department of Clinical Pharmacy, College of Pharmacy, Jazan University, Jazan, Saudi Arabia; 2 Pharmacy Practice Research Unit, College of Pharmacy, Jazan University, Jazan, Saudi Arabia; 3 Department of Psychology, College of Education, Jazan University, Jazan, Saudi Arabia; 4 Mohammed bin Nasser Hospital, Ministry of Health, Jazan, Saudi Arabia; University of Southern Queensland, AUSTRALIA

## Abstract

**Objectives:**

This study objectives were to investigate maternal psychological distress, mothers’ fear of their children contracting COVID-19, mothers’ perceptions of the information available regarding children and COVID-19, changes in children’s behavior during lockdown, and concerns of pregnant women in Saudi Arabia.

**Methods:**

This cross-sectional study surveyed women aged 18 years and older who either had children under 10 years of age or were pregnant at the time of the survey. The outcomes included psychological distress, mothers’ fear of their children contracting COVID-19, change in children’s behaviors during COVID-19 lockdown and pregnant women’s concerns. Multivariable ordinary least squares regression models were employed to examine the adjusted associations between sociodemographic factors and psychological distress, as well as fear of COVID-19.

**Results:**

Of 628 women, 11.8% (*n* = 74) were pregnant at the time of survey. Most of the pregnant women (89.2%, *n* = 66) had some degree of concerns about their unborn babies getting infected during delivery in the hospital. Among mothers of children under 10 years of age (*n* = 564), half (*n* = 282) reported change in their children’s behavior during the lockdown. Most mothers and pregnant women (94.9%, *n* = 569) had some degree of psychological distress. Mothers and pregnant women with a college degree had significantly lower psychological distress (β = -1.346; *p* = 0.014) than women with a high school education or less. Similarly, mothers and pregnant women with monthly family income ≥ US$ 1,333 had lower psychological distress than those with < US$ 1,333. Women with pre-existing chronic physical (β = 2.424; p < 0.001) or mental (β = 4.733; p < 0.001) conditions had higher psychological distress than those without these conditions. Having children in the house was a contributory factor for higher psychological distress. For example, mothers with one child (β = 2.602; p = 0.007) had significantly higher psychological distress compared to expectant mothers without children in the house.

**Conclusions:**

Most mothers and expectant mothers in our study had moderate to high levels of psychological distress during the COVID-19 pandemic outbreak in Saudi Arabia. Education, family income and chronic mental and physical conditions were associated with high psychological distress in Saudi Arabia during COVID-19.

## Introduction

The novel coronavirus disease 2019 (COVID-19) emerged in Wuhan, China, and spread to 217 countries [1]. The World Health Organization declared it a global pandemic on March 11, 2020 [2]. Billions of people around the world have been affected as countries implemented measures to fight the pandemic, among them Saudi Arabia. Prior to May 9, 2021, Saudi Arabia had recorded 425,442 confirmed cases of COVID-19 and 7,059 deaths had been registered [3]. The COVID-19 pandemic has had undesirable physical and psychological consequences affecting societies, families and individuals [4, 5].

From a family perspective, the mother plays an important role as the primary caregiver for the children. Maternal mental health is a matter of serious concern, as females are reported to be at a higher risk for anxiety and depression [4, 6]. The implementation of measures to contain COVID-19 was estimated to negatively affect psychosocial family functioning and may have escalated the risk of depression among mothers [6].

Recent socioeconomic changes in Saudi Arabia, including increased education and employment among women [7], has increased burdens on women, putting them at greater risk for developing psychological stress. During social isolation In Italy, mothers self-reported that they experienced increased frustration and sadness [8]. Abrupt deviations from the normal schedule, such as closing of schools and childcare facilities, work from home and imposed lockdowns, have resulted in feelings of helplessness and abandonment in women [9]. Studies on psychological distress, especially among women prior to COVID-19, are scarce in Saudi Arabia. However, one study in Saudi Arabia indicated that the prevalence rates of depression and general anxiety during the COVID-19 period were higher than the rates prior to the pandemic [10]. Furthermore, two studies conducted during the pandemic found that women in general had higher psychological distress than men in Saudi Arabia [11, 12]. Therefore, our intention was to quantify levels of psychological distress among mothers and expectant mothers during the COVID-19 pandemic as a baseline reference for future research.

In addition to mothers with young children, pregnant women may be exposed to added causes of psychological distress, such as the health and safety of their unborn child [4]. Women who are pregnant can be at elevated risk of moderate to severe anxiety due to the stress of preparing for delivery and fear of COVID-19 infection to the baby and themselves [13]. As compared to reports before the COVID-19 pandemic, pregnant women during the pandemic reported that they had increased levels of anxiety and depression [14].

Although children are at a lower risk of infection, COVID-19 has impacted them due to closure of schools and restrictions from outdoor spaces [15]. These measures have directly or indirectly affected the psychosocial functioning of the family, which can increase the risk of maternal psychological distress. During the pandemic, mothers have reported a negative change in their children’s behaviors, including lack of discipline and hyperactivity [8]. The pandemic situation has therefore increased the probability of maternal psychological distress, and its negative outcomes may also place children at risk [16].

Due to the above concerns, this study was undertaken to investigate maternal psychological distress, mothers’ fear of their children contracting COVID-19, mothers’ perceptions of the information available regarding children and COVID-19, changes in children’s behavior during lockdown, and concerns of pregnant women in Saudi Arabia. Furthermore, we assessed the relationships between demographics, socioeconomic status, chronic physical and mental conditions, and the main outcomes (psychological distress and mothers’ fear of their children contracting COVID-19).

## Methods

### Participants and procedure

This study used a cross-sectional, questionnaire-based design. The sample comprised women aged 18 years and older who either had a child under 10 years of age or were pregnant. The Institutional Research Review and Ethics Committee at Jazan University reviewed and approved this study (IRB No. REC41/9Al002). Our sample size was determined based on the total number of married, divorced and widowed women in Saudi Arabia [17]. The required sample size with efficient statistical power was calculated using Raosoft® online sample size calculator [18]. The calculation yielded a sample size of 385 based on a population of 4,585,017 women, a 5% margin of error, a 95% confidence level, and a 50% response distribution. To ensure the study’s accuracy and power, the sample size was increased to 628 women.

The questionnaire was developed after an extensive literature review to identify existing instruments and scales. An initial draft of the questionnaire was developed with the following sections: 1) demographics; 2) maternal psychological distress and concerns; 3) mothers’ fear of their children contracting COVID-19; and 4) change in children’s behavior during COVID-19 lockdown. After receiving ethical approval, cognitive interviews were conducted with 10 mothers of children under 10 years of age in Saudi Arabia to examine the face validity of the questionnaire.

**Fig 1:**
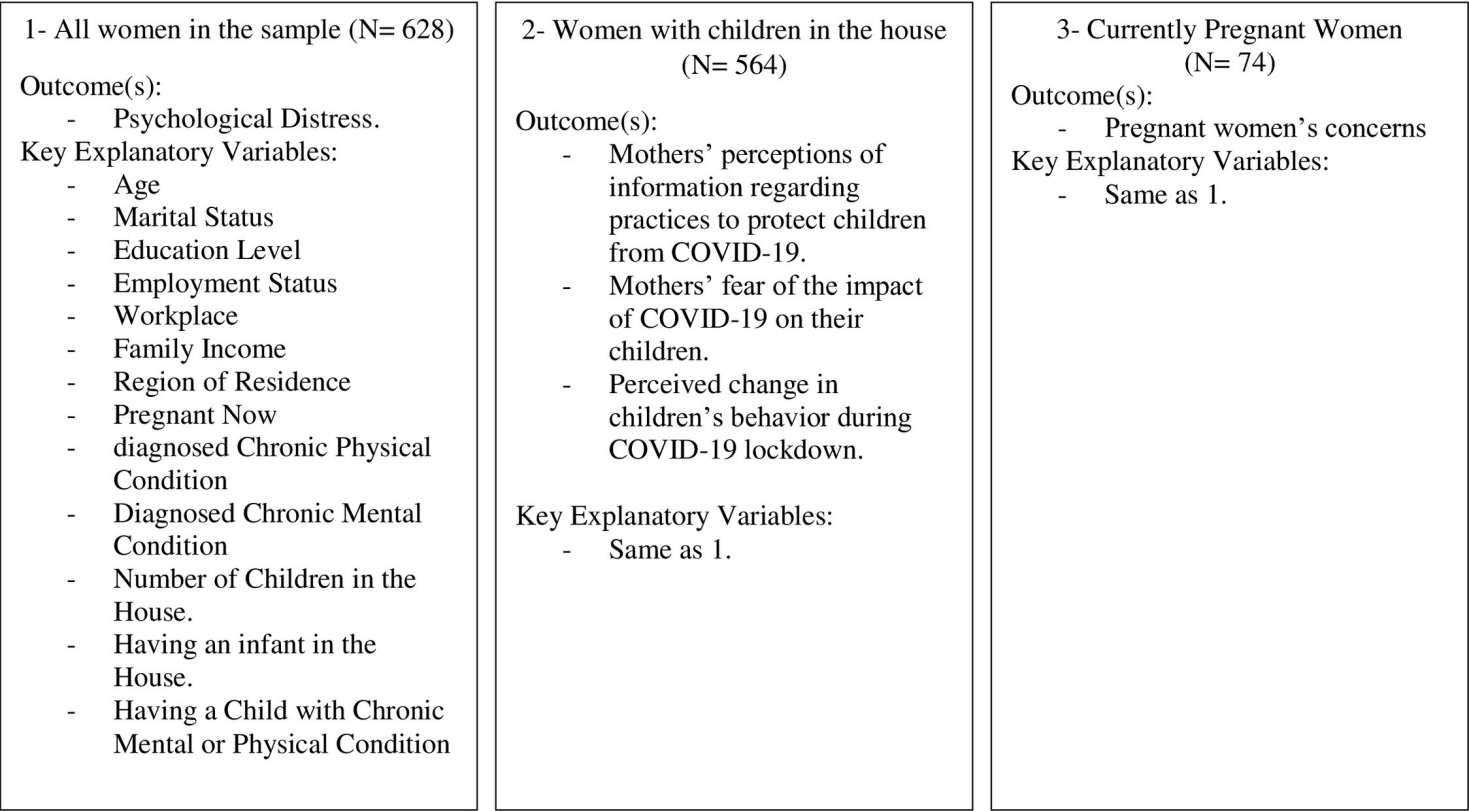
Study’s sample, subsamples, and measured variables.

The study was conducted from July to August 2020. Due to the pandemic social distancing measures, the self-report questionnaire was sent to potential respondents as an online Google Form link. The snowball sampling method was used to invite respondents. Initially, 10 participants were selected to ensure a broad representation of age, occupation, education level, and location. This set of respondents then forwarded the questionnaire to 10 acquaintances whom they considered suitable for the survey, and this second set forwarded the questionnaire in the same way. Participants were required to provide online informed consent prior to answering the questionnaire and were informed that they were free to withdraw from the study at any time. The final sample included 628 women who were either mothers of children under 10 years of age or were pregnant. Fig 1 shows the study’s sample, subsamples, and measured variables.

### Measures

#### Outcomes

1—Psychological Distress: Psychological distress was measured using the 6-item Kessler-6 Non-Specific Psychological Distress Scale (K6) [19], which was translated from English to Arabic by experienced language experts [20]. This measure has been validated among Arab populations [21]. The K6 included the following questions: “In the past 30 days, about how often did you feel: (1) so sad nothing could cheer you up? (2) nervous? (3) restless or fidgety? (4) hopeless? (5) that everything was an effort? (6) worthless?” Participants responded to these questions using a Likert-type scale ranging from 0 (*none of the time*) to 4 (*all of the time*). The K6 summary score ranges from 0–24 with higher scores representing higher psychological distress. Following a previously published study [22], we categorized women in our sample into four groups: 1) no psychological distress (0 score); 2) low psychological distress (1–5 score); 3) moderate psychological distress (6–10 score); and 4) high psychological distress (11–24 score).

2—Mothers’ fear of their children contracting COVID-19: We measured mothers’ fear of their children becoming infected using the validated Arabic version of the 7-item Fear of COVID-19 scale [23]. Participants were asked to consider the risk of COVID-19 to their child/children and to indicate their agreement/disagreement using a 5-point scale ranging from 0 (*strongly disagree*) to 5 (*strongly agree*) with the following statements: 1) I am most afraid of COVID-19; 2) It makes me uncomfortable to think about COVID-19; 3) My hands become clammy when I think about COVID-19; 4) I am afraid of losing my child/children because of COVID-19; 5) When watching news and stories about COVID-19 on social media, I become nervous or anxious; 6) I cannot sleep because I’m worrying about my child/children getting COVID-19; and 7) My heart races or palpitates when I think about my child getting COVID-19. Scores on each item were added to provide a summary score of mothers’ fear of their children contracting COVID-19 ranging from 0–35, with higher scores representing higher levels of fear.

3—Information available for mothers regarding children and COVID-19: We measured mothers’ perception of the information available about practices to protect children from COVID-19. Mothers were asked to consider the information related to children during the COVID-19 pandemic, and to indicate their agreement/disagreement on a 5-point scale from *strongly disagree* to *strongly agree* on the following statements: 1) Clear guidelines are publicly available for precautionary measures to be followed by children during COVID-19; 2) Children aged less than 10 years are not able to follow precautionary measures by themselves; 3) Children of all ages should wear face masks when going outdoors; 4) Face masks for children must be made available in community pharmacies.

4—Change in children’s behaviors during COVID-19 lockdown: We asked mothers if their children experienced one or more of the following behaviors during COVID-19 lockdown: 1) Excessive crying; 2) Irritability; 3) Returning to behaviors they had outgrown (for example, toileting accidents or bedwetting); 4) Unhealthy eating habits; 5) Unhealthy sleeping habits; 6) Non-cooperative; 7) Difficulty with attention and concentration.

5—Pregnant women’s concerns: Pregnant women’s concerns about their unborn children and COVID-19 was measured with the question: “Are you concerned that your child may get infected with COVID-19 during the delivery or during your stay at the hospital?” Responses were provided on a scale of 1 to 5, from 1 (*not at all concerned*) to 5 (*extremely concerned*).

#### Explanatory variables

The selection of explanatory variables was guided by Andersen’s Expanded Behavioral Model [24]. According to this model, health outcomes may be influenced by predisposing factors (age and sex), enabling factors (education level and economic status), need factors (mental and physical health) and external factors (region of residence). The explanatory variables in this study included mothers’ demographics, such as age (in years), marital status (married and widowed/divorced), education (less than high school to college and post-graduate studies), employment status (employed, unemployed/seeking job, stay at home, student), monthly family income (< US$ 1,333; US$1,333–2,667; > US$ 2,667), presence of chronic mental conditions (yes, no), presence of chronic physical conditions (yes, no), currently pregnant (yes, no) and region of residence (North, East, Mid, West, South). We also measured other information regarding children, including number of children in the house, having infants in the house, and having a child with a chronic mental or physical condition.

### Statistical techniques

The data were downloaded as a Microsoft Excel sheet from the Google Forms survey page, and all the responses were anonymized and coded. Frequency and percentages were calculated and reported for categorical variables. Means and standard deviations were computed for continuous variables. Internal reliability for the mothers’ fear of their children contracting COVID-19 and general psychological distress scales were tested using Cronbach’s alpha. Ordinary least squares regressions with heteroskedasticity-robust standard errors option were conducted to examine the adjusted relationships between the explanatory variables and outcomes (psychological distress and mothers’ fear of their children contracting COVID-19 scales). The final model where the outcome was psychological distress included age, marital status, education, employment status, family income, the presence of chronic mental condition, the presence of chronic physical condition, number of children in the house, pregnancy status, and having a child with chronic mental or physical condition. The final model where the outcome was mothers’ fear of their children contracting COVID-19 included age, marital status, education level, employment status, family income, number of children in the house, pregnancy status, and having a child with a chronic mental or physical condition. All statistical analyses were performed using Stata 15.0 (Stata Corp LP, College Station, USA).

## Results

### Description of the study sample

The study sample consisted of 628 women who were either mothers of a child/children under 10 years of age or were pregnant. The average age of women in our sample was 31.8 years (*SD* = 7.2). Of the sample, 95.2% were married, 70.1% had at least a college degree and 32.3% were employed. Among those employed, 73.4% worked in a university/school, 15.8% in a hospital, and 10.8% in office/malls. The majority of the sample had a monthly family income of US$ 2,667 or more. At the time of the survey, 11.8% (*n* = 74) were pregnant. Additionally, 11.6% of the women in the sample had a diagnosed chronic physical condition. Most mothers had more than one child in the house, while 15.9% had a child with chronic mental or physical conditions. Table 1 depicts the characteristics of the study sample.

**Table 1:** Descriptive statistics of mothers and pregnant women in the study sample (*N* = 628).

Baseline characteristic	*N*	*%*
**Marital Status**		
Married	598	95.2
Widow/Divorced	30	4.8
**Education**		
≤ High School	188	29.9
College, +	440	70.1
**Employment Status**		
Employed	203	32.3
Unemployed/Looking for a job	155	24.7
Stay at home	198	31.5
Student	72	11.5
**Workplace**		
Hospital	32	15.8
University/School	149	73.4
Office/Mall	22	10.8
**Family Income**		
<1,333 US$	144	23.0
1,333–2,667 US$	237	37.7
>2,667 US$	247	39.3
**Region of Residence**		
North	18	2.9
East	40	6.4
West	104	16.6
Mid	102	16.2
South	364	58
**Currently Pregnant**		
No	553	88.2
Yes	74	11.8
**Having a diagnosed Chronic Physical Condition**		
No	555	88.4
Yes	73	11.6
**Having a diagnosed Chronic Mental Condition**		
No	604	96.2
Yes	24	3.8
**Number of Children in the House**		
No Children	36	5.7
One Child	213	33.9
2 children	226	36
≥ 3 Children	153	24.4
**Having an infant in the House**		
No	530	84.4
Yes	98	15.6
**Having a Child with Chronic Mental or Physical Condition**		
No	528	84.1
Yes	100	15.9
**Psychological Distress**		
No Distress (0 score)	32	5.1
Low Distress (1–5 score)	133	21.2
Moderate Distress (6–10 score)	218	34.7
High Distress (11–24 score)	245	39

### General psychological distress among mothers and expectant mothers

Table 1 shows mothers’ and pregnant women’s (*N* = 628) psychological distress during the time of the COVID-19 outbreak in Saudi Arabia. The mean psychological distress score was 9.2 (*SD* = 5.2). The Cronbach’s alpha for the scale was 0.85. The majority (94.9%) of the sample self-reported some degree of psychological distress during the time of the survey. Specifically, 34.7% reported moderate psychological distress, and 39% reported high psychological distress.

### Explanatory variables and psychological distress

Table 2 displays parameter estimates of the explanatory variables obtained by ordinary least squares regression on psychological distress. In the adjusted analyses, no relationship was found between marital status, employment status, and psychological distress. However, a significant relationship was identified between education level and psychological distress. Mothers and expectant mothers with a college degree or higher had lower levels of psychological distress (β = -1.346; *p* = 0.014) than those with a high school education or less. Likewise, mothers and expectant mothers with high family incomes had lower levels of psychological distress than those with lower family incomes. Specifically, mothers and expectant mothers with monthly family income of US$ 1,333–2,667 had lower levels of psychological distress (β = -1.376; *p* = 0.011) than their counterparts with < US$ 1,333 family income. Similarly, women with more than US$ 2,667 family income had lower levels of psychological distress (β = -1.370; *p* = 0.014) than those with family income of < US$ 1,333.

**Table 2:** Parameter estimates of the explanatory variables from ordinary least squares regression outcome relative to psychological distress. Mothers, and pregnant women (*N* = 628).

	Unadjusted			Adjusted			
Explanatory Variable	β	95% Confidence Interval	*p-value*	β	95% Confidence Interval	β Standardized	*p-value*
**Age**	-0.094	(-0.154 –-0.034)	0.002	-0.073	(-0.15–0.003)	-0.099	0.06
**Marital Status**							
Married	Reference						
Widow/Divorced	0.211	(-1.649–2.071)	0.824	0.045	(-1.729–1.819)	0.002	
**Education**							
≤ High School	Reference						
College, +	-1.158	(-2.101 –-0.216)	0.016	-1.346	(-2.416 –-0.275)	-0.118	0.014
**Employment Status**							
Employed	Reference						
Unemployed	0.972	(-0.105–2.049)	0.077	0.091	(-1.088–1.269)	0.008	0.88
Stay at home	0.85	(-0.179–1.88)	0.105	-0.782	(-1.975–0.411)	-0.07	0.199
Student	1.902	(0.483–3.321)	0.009	1.131	(-0.561–2.822)	0.069	0.19
**Family Income**							
<1,333 US$	Reference						
1,333–2,667 US$	-1.611	(-2.695 –-0.527)	0.004	-1.376	(-2.431 –-0.32)	-0.128	0.011
>2,667 US$	-1.797	(-2.886 –-0.709)	0.001	-1.370	(-2.465 –-0.276)	-0.128	0.014
**Having a diagnosed Chronic Physical Condition**							
No	Reference						
Yes	1.619	(0.278–2.96)	0.018	2.424	(1.154–3.694)	0.149	<0.001
**Having a diagnosed Chronic Mental Condition**							
No	Reference						
Yes	4.732	(2.515–6.949)	<0.001	4.733	(2.657–6.808)	0.174	<0.001
**Number of Children in the House**							
No children	Reference						
One child	1.011	(-0.95–2.971)	0.312	2.602	(0.701–4.503)	0.235	0.007
2 children	1.452	(-0.508–3.412)	0.146	2.965	(0.977–4.952)	0.272	0.004
≥ 3 children	1.028	(-0.997–3.053)	0.319	2.524	(0.519–4.528)	0.207	0.014
**Currently Pregnant**							
No	Reference						
Yes	0.662	(-0.585–1.908)	0.298	0.721	(-0.545–1.987)	0.045	0.264
**Having a Child with Chronic Mental or Physical Condition**							
No	Reference						
Yes	0.655	(-0.492–1.803)	0.263	0.337	(-0.794–1.467)	0.024	0.559

On the other hand, women in our sample with a diagnosed chronic physical condition had significantly higher psychological distress (β = 2.424; *p* < 0.001) than those without a chronic physical condition. Similarly, mothers and expectant mothers with a diagnosed chronic mental condition had higher psychological distress (β = 4.733; *p* < 0.001) than those without a chronic mental condition. In the adjusted analysis, psychological distress increased as number of children increased. Mothers with one child (β = 2.602; *p* = 0.007), two children (β = 2.965; *p* = 0.004), or three or more children (β = 2.524; *p* = 0.014) had significantly higher psychological distress compared to expectant mothers without children in the house.

### Information about children during the COVID-19 pandemic

**Fig 2:**
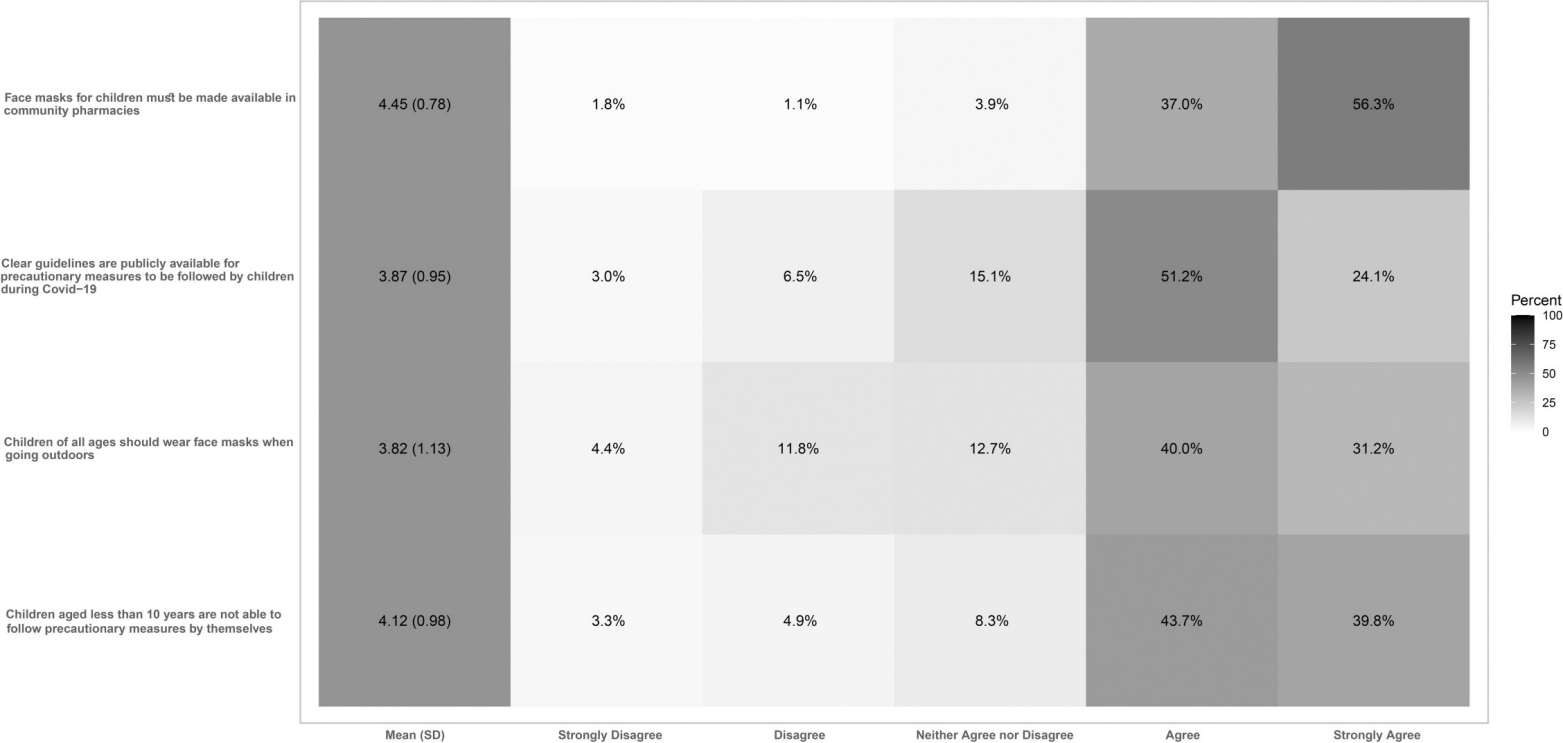
Mothers’ (n = 564) perceptions of information regarding practices to protect children from COVID-19.

Fig 2 displays the results regarding mothers’ (*n* = 564) perceptions of information regarding practices to protect children from COVID-19. Of the study sample, 75% either agreed or strongly agreed to the statement “Clear guidelines are publicly available for precautionary measures to be followed by children during COVID-19.” Additionally, 83% either agreed or strongly agreed to the statement “Children aged less than 10 years are not able to follow precautionary measures by themselves.” The majority of mothers (71%) either agreed or strongly agreed that children of all ages should wear face masks when going outdoors. Finally, 93% either agreed or strongly agreed that face masks for children must be made available at community pharmacies.

### Perceived change in children’s behavior during COVID-19 lockdown

**Fig 3:**
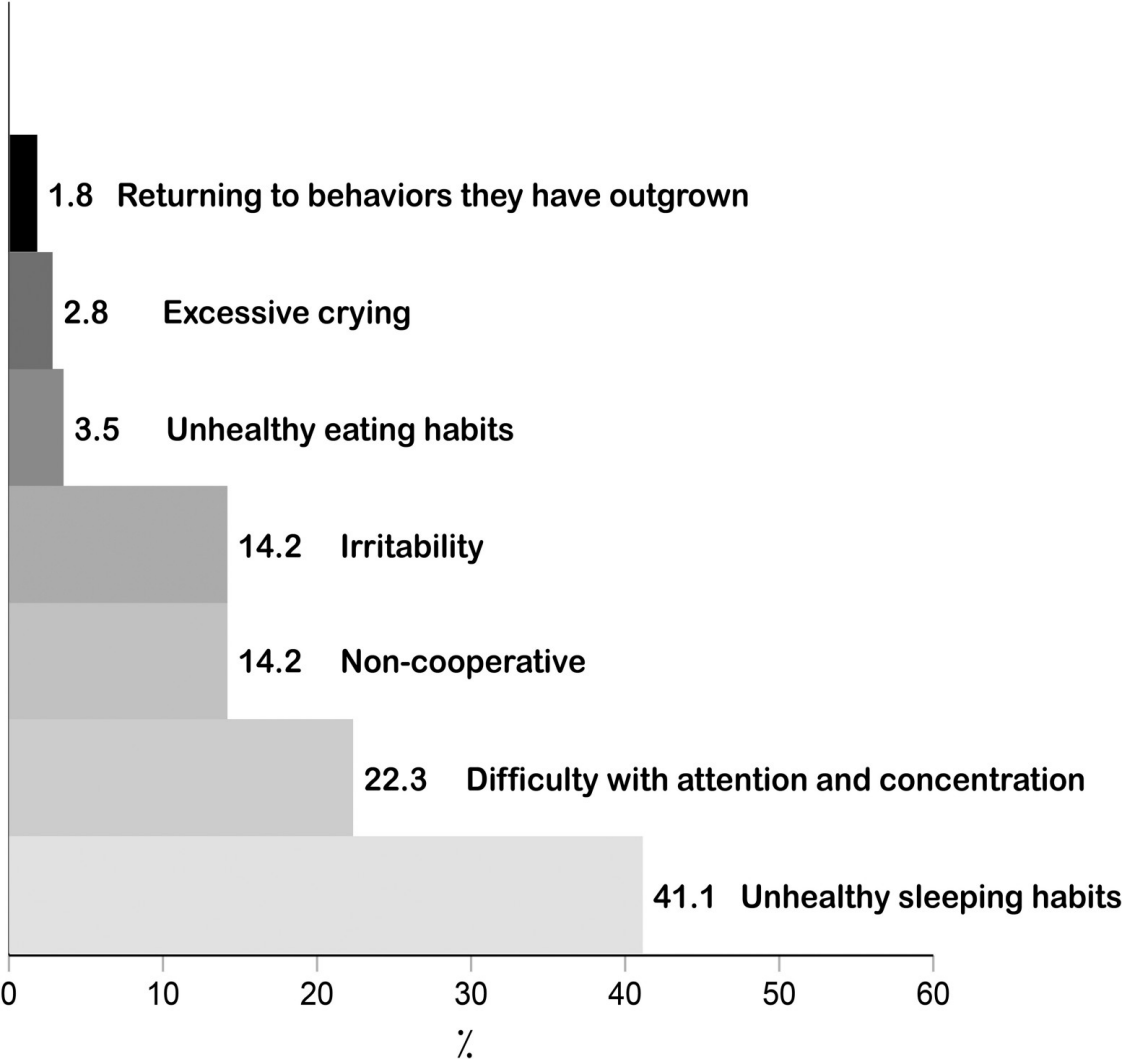
Perceived change in children’s behavior during COVID-19 lockdown (n = 564).

Fig 3 displays mothers’ (*n* = 564) reported change in their children’s behaviors during the COVID-19 lockdown. Among mothers of children under 10 years of age, 49.6% reported a change in their children’s behaviour during lockdown. The most reported behavioral change was unhealthy sleeping habits (41.1%), followed by difficulty with attention and concentration (22.43%), non-cooperative (14.2%) irritability (14.2%), and unhealthy eating habits (3.5%). Furthermore, 1.8% of mothers reported that their children had returned to behaviors they had outgrown such as toileting accidents or bedwetting.

### Mothers’ fear of their children contracting COVID-19

The mean score for mother’s (*n =* 564) fear of their children contracting COVID-19 was 21.1 (*SD* = 6.1). The Cronbach’s alpha for the scale was 0.87. Table 3 displays unadjusted and adjusted relationships between the explanatory variables and mothers’ fear of their children contracting COVID-19. In the adjusted analysis, no significant relationship was found between age, marital status, employment status and mothers’ fear of their children contracting COVID-19. Nevertheless, mothers with a higher level of education (a college degree or post-graduate studies) had a lower fear of their children contracting COVID-19 (β = -2.658; *p* < 0.001) than those with a high school education or less. Furthermore, mothers with high family incomes had lower fear of their children contracting COVID-19 than mothers with lower family incomes. Mothers with family income of US$ 1,333–2,667 had significantly lower fear of their children contracting COVID-19 (β = -2.016; *p* = 0.005) than mothers with family income of < US$ 1,333. Likewise, mothers with family incomes more than US$ 2,667 had lower fear of their children contracting COVID-19 (β = -2.341; *p* = 0.001) than those with family income of < US$ 1,333.

**Table 3:** Parameter estimates of the explanatory variables from ordinary least squares regression outcome relative to mothers’ fear of their children contracting COVID-19 (*n* = 564).

	Unadjusted			Adjusted			
Explanatory Variable	β	95% Confidence Interval	*p-value*	β	95% Confidence Interval	β Standardized	*p-value*
**Age**	0.051	(-0.031–0.133)	0.219	0.083	(-0.017–0.183)	0.09	0.103
**Marital Status**							
Married	Reference						
Widow/Divorced	-0.288	(-2.512–1.937)	0.8	-1.559	(-3.575–0.458)	-0.054	0.13
**Education**							
≤ High School	Reference						
College, +	-2.224	(-3.314 –-1.134)	0	-2.658	(-3.98 –-1.335)	-0.197	<0.001
**Employment Status**							
Employed	Reference						
Unemployed	0.372	(-0.956–1.699)	0.583	0.172	(-1.24–1.584)	0.012	0.811
Stay at home	0.346	(-0.924–1.616)	0.593	-1.312	(-2.846–0.222)	-0.101	0.094
Student	-1.033	(-2.754–0.689)	0.239	-0.263	(-2.278–1.751)	-0.014	0.797
**Family Income**							
<1,333 US$	Reference						
1,333–2,667 US$	-2.047	(-3.386 –-0.709)	0.003	-2.016	(-3.408 –-0.624)	-0.161	0.005
>2,667 US$	-2.368	(-3.667 –-1.069)	0	-2.341	(-3.723 –-0.96)	-0.19	0.001
**Number of Children in the House**							
One child	Reference						
2 children	0.241	(-0.979–1.46)	0.698	0.267	(-0.942–1.476)	0.022	0.664
≥ 3 children	0.255	(-0.998–1.508)	0.69	0.048	(-1.228–1.325)	0.004	0.941
**Currently Pregnant**							
No	Reference						
Yes	-0.372	(-2.073–1.329)	0.668	-0.21	(-1.957–1.536)	-0.01	0.813
**Having a Child with Chronic Mental or Physical Condition**							
No	Reference						
Yes	1.475	(0.054–2.897)	0.042	1.17	(-0.202–2.541)	0.072	0.094

### Pregnant women’s concerns about COVID-19 and their children

**Fig 4:**
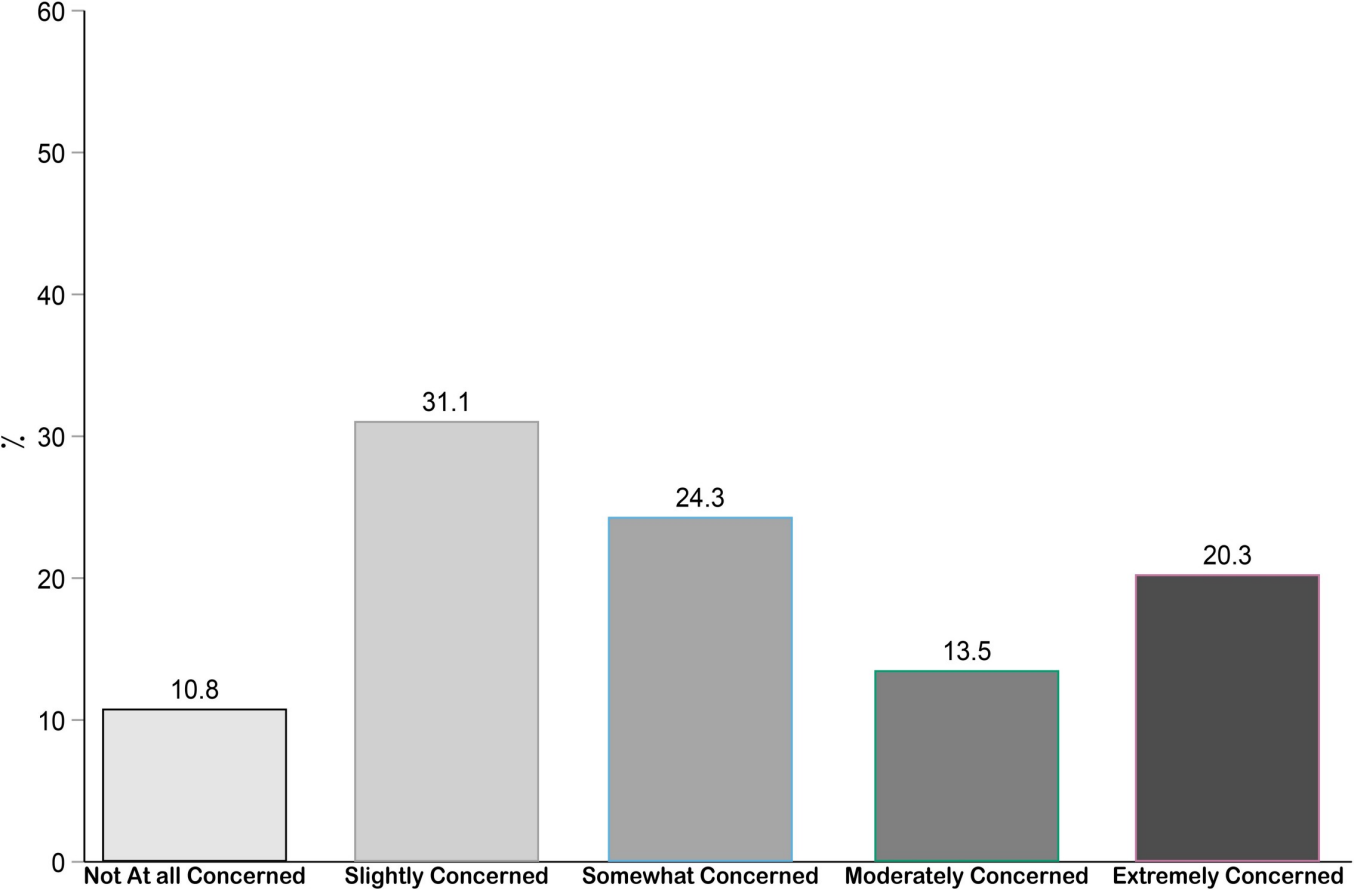
Concerns of pregnant women (*N* = 74) about the risk for their children contracting COVID-19 during delivery in the hospital.

Fig 4 illustrates pregnant women’s (*n* = 74) concerns about the risk for their children of contracting COVID-19 during delivery in the hospital. The majority (89.2%) of pregnant women had some degree of concerns. Among the pregnant women, 31.1% were slightly concerned, 24.3% were somewhat concerned, 13.5% were moderately concerned and 20.3% were extremely concerned about their unborn babies getting infected with COVID-19 during delivery in the hospital.

## Discussion

The current study examined the psychological distress of mothers of children under 10 years of age as well as pregnant women in Saudi Arabia during the COVID-19 pandemic outbreak. The study also examined mothers’ fear of their children contracting COVID-19. The results showed that most of the mothers and pregnant women in the study sample had some degree of psychological distress, and the majority had either moderate or high levels of psychological distress. Previous studies found that women and pregnant women worldwide experienced higher psychological distress during the COVID-19 pandemic [8, 25–27]. Our results are consistent with the evidence of the effect of COVID-19 on the mental health of women and pregnant women [8, 25–27]. Pregnant women are a vulnerable population who experience both physiological and hormonal changes. Obstetricians and other healthcare professionals must closely monitor the mental health of pregnant women in this era of COVID-19 in Saudi Arabia and worldwide.

Previous studies found that mental health interventions and support programs have improved mental health of the participants during medical crises [28–30]. Psychological, psychiatric, education and other interventions either in person or online have improved the mental health of individuals during COVID-19, Ebola, and SARS pandemics [29–31]. Nevertheless, more studies are required to assess the effectiveness of these interventions. Furthermore, universal digital mental health interventions are crucial to address the mental health of the population during lockdown and other restrictions [31].

The study results indicated that socioeconomic status was inversely associated with psychological distress among mothers and pregnant women. Specifically, higher family incomes were associated with lower psychological distress. This finding is consistent with previous reports showing that high socioeconomic status was associated with lower psychological distress and depression rates. For instance, a study in Sweden found that women with low family incomes were more likely to have higher psychological distress [32]. Furthermore, we found that education was inversely associated with psychological distress. Similarly, a Canadian study found an association between lower family income and lower education with COVID-19-related depression in mothers with children under the age of 18 months [6]. In general, socioeconomic status is a strong predictor of perceived physical health [33], mental health [34] and mortality [35]. This relationship between socioeconomic status and mental health was supported among mothers and pregnant women in Saudi Arabia.

We also found that mothers and pregnant women with a diagnosed chronic physical condition had higher psychological distress than those without. Chronic physical conditions increase individuals’ risk of severe illness from COVID-19 [36]. Also, having a pre-existing chronic mental condition among mothers and expectant mothers was associated with higher psychological distress. These findings were also consistent with the previously mentioned Canadian study, in which having a history of mental illness was associated with COVID-19- related depression in mothers with children under the age of 18 months [6]. Psychological distress and poor mental health were associated with negative health outcomes [37] among adults with chronic physical conditions. Therefore, it is crucial to address poor mental health among women in Saudi Arabia.

We also explored mothers’ awareness of COVID-19 information related to children. Based on the responses, participants did not have clear awareness of the information related to the use of facemasks in children. This was evident from the majority of respondents’ agreement that children of all ages should wear face masks when going outdoors. The Saudi Arabia Ministry of Health has issued publicly available COVID-19 guidelines for children [38]. These guidelines are in line with the recommendation by the Centers for Disease Control and Prevention (CDC) that facemasks should not be worn by children under 2 years of age [39].

Moreover, the study results indicated that mothers of children under 10 years of age had high levels of fear of their children contracting COVID-19. Furthermore, mothers with a higher number of children had higher psychological distress. Taken together, these findings indicate that COVID-19 puts pressure on mothers in Saudi Arabia. Additionally, one-third of pregnant women had moderate or extreme concerns about their newborn child getting infected in the hospital during birth. Similar findings were reported by an American study, which concluded that expectant mothers who feared infection to themselves and to their child during birth had an increased risk of experiencing moderate or severe anxiety [13]. It is evident that expectant mothers are under additional prenatal stress, as they are worried about the safety of their child as well as themselves.

Furthermore, almost 50% of the mothers in the sample experienced a change in their children’s behaviors during the COVID-19 lockdown. In our study, unhealthy sleeping habits were the most notable behavior observed by mothers in their children, Additionally, around one-fourth of mothers found their children having difficulty with attention and concentration. These observations are consistent with a Chinese study that investigated behavioral disorders in children during the pandemic, and reported sleep disturbances, attention difficulties and agitation [40]. Change in children’s behaviors could be due to the long lockdown they experienced, social distancing or online schooling. As mothers are the primary caregivers for children in Saudi Arabia, our results specifically highlight the impact of COVID-19 on mothers and children.

The present study had some limitations. Since this is a cross-sectional study, we were not able to assess the temporal associations between the explanatory variables and the outcomes of this study. Our results may not be generalizable to all mothers and pregnant women in Saudi Arabia, as most of the women in the sample were young and highly educated. This may be due to the study being conducted through an online questionnaire owing to the lockdown and other precautionary measures. This study is prone to sampling and response bias as it is based on an online questionnaire. Additionally, we did not measure and control other social factors such as family cohesion and social support. However, this study has several advantages. To the best of the authors’ knowledge, this is the first study to measure psychological distress among Saudi mothers. In addition, we had a relatively large sample size and we measured and controlled for many factors that may affect our outcomes including predisposing, enabling, need and external factors. Finally, we included mothers from different regions within Saudi Arabia.

## Conclusion

Most mothers and expectant mothers in our study had moderate to high levels of psychological distress during the COVID-19 pandemic outbreak in Saudi Arabia. Furthermore, most mothers and expectant mothers had fears or concerns about their children getting infected with COVID-19. Education, family income and chronic mental and physical conditions were associated with high psychological distress in Saudi Arabia during COVID-19.

## Supporting information

S1 Data (XLS)
